# Fabrication of poly ($\varepsilon$-caprolactone) 3D scaffolds with controllable porosity using ultrasound

**DOI:** 10.1038/s41598-025-06818-9

**Published:** 2025-07-02

**Authors:** Martin Weber, Dmitry Nikolaev, Mikko Koskenniemi, Jere Hyvönen, Joel Jääskeläinen, Armand Navarre, Ekaterina Takmakova, Arun Teotia, Pekka Katajisto, Robert Luxenhofer, Edward Hæggström, Ari Salmi

**Affiliations:** 1 Electronics Research Laboratory, Department of Physics, Faculty of Science, University of Helsinki, Helsinki, Finland; 2 Technical college of Blois, Blois, France; 3 Soft Matter Chemistry, Department of Chemistry, and Helsinki Institute of Sustainability Science, Faculty of Science, University of Helsinki, Helsinki, Finland; 4 Institute of Biotechnology (HiLIFE), Faculty of Biological and Environmental Sciences, University of Helsinki, Helsinki, Finland

**Keywords:** Acoustics, Biomedical materials

## Abstract

3D printing has progressed significantly, allowing objects to be produced using a wide variety of materials. Recent advances have employed focused ultrasound in 3D printing, to allow printing inside acoustically transparent materials. Here we introduce a selective ultrasonic melting (SUM) method for 3D printing of poly ($\varepsilon$-caprolactone) powder mixed with water. The printing was done by mechanically moving a focused ultrasound transducer. The microstructure and porosity of the prints were analyzed with micro-computed tomography. The open porosity of the printed samples was determined using the water intrusion method and by passing fluorescent microspheres through the structure. The cytocompatibility of the printed structures was confirmed by seeding NIH-3T3 fibroblast cells on the scaffolds, followed by analysis using live/dead fluorescent assay and visualization using scanning electron microscopy. We demonstrated that SUM is a viable technique to print structures with active control of their porosity. This method provides an alternative to methods such as fused deposition modelling and material jetting.

## Introduction

Additive manufacturing can be grouped in 7 classes according to ASTM^1^ and several of them construct objects by melting powders composed of submillimeter particles, such as selective laser sintering (SLS), selective laser melting (SLM), and electron beam melting (EBM)^2^. These methods use a focused laser or electron beam to heat the material and fuse the powder particles. It is applicable for the fabrication of biomimetic structures with small feature size^3^ and the fabrication of porous structures^4^.

Since neither the laser nor the electron beams can penetrate deeply into the powder, the printing is performed layer-by-layer with additional powder applied between the processing of each layer. Another emerging approach involves the use of focused ultrasound (FUS), which can penetrate a wide range of materials and concentrate acoustic energy precisely within a desired region. This enables the printing inside of existing structures and printing below the surface of the powder material. This differentiates the method from conventional powder bed fusion and direct energy deposition, where the printing can only be performed at the surface layer.

FUS can rapidly cure silicone polymers via sonochemical reactions^5^, and holographic sound printing enables cross-sectional polymerisation of silicone elastomers^6^. FUS can initiate polymerization reactions of self-enhancing sonicated ink within deep layers of tissue via sonothermal-induced gelation^7,8^. A mixture of natural products such as egg white and potato flour can be sonothermally solidified and used to print 3D objects^9^. Acoustic radiation force can create 3D objects through particle assembly and material structuring which includes the use of acoustic holograms in liquid^10^ and acoustic field control with phased arrays in air^11^.

3D printing promises use in personalized medical applications, from implants and prosthetics to tissue-engineered structures for tissue regeneration^12,13^. Notably, porous structures that mimic extracellular matrices are crucial in bone tissue engineering^14–17^ and in drug delivery systems^18,19^. PCL is widely used in these applications, offering advantages such as biodegradability, processability, biocompatibility, and robust mechanical properties^18,20–22^.

Due to the thermoplastic properties of PCL, the most common printing technique is fused deposition modelling (FDM)^23,24^ which allows printing cell-loaded scaffolds with controlled porosity^25^. This method suffers from low resolution, manufacturing complexity, and unsuitability for *in situ* printing.

We introduce the selective ultrasonic melting (SUM) method that uses FUS to fuse PCL powder mixed with water into solid structures. In this technique, the ultrasonic beam penetrates the PCL sample, releasing thermal energy in the focus, which causes the PCL to melt, forming a voxel. By moving the focal spot within the sample, a porous 3D structure is formed.

We used the NIH-3T3 fibroblast cell line to verify the cytocompatibility of structures printed at different feed rates. Cell viability was determined using live/dead fluorescent imaging. Furthermore, the cell-laden scaffolds were visualized by SEM imaging.

## Results

### Printed samples

**Fig. 1:**
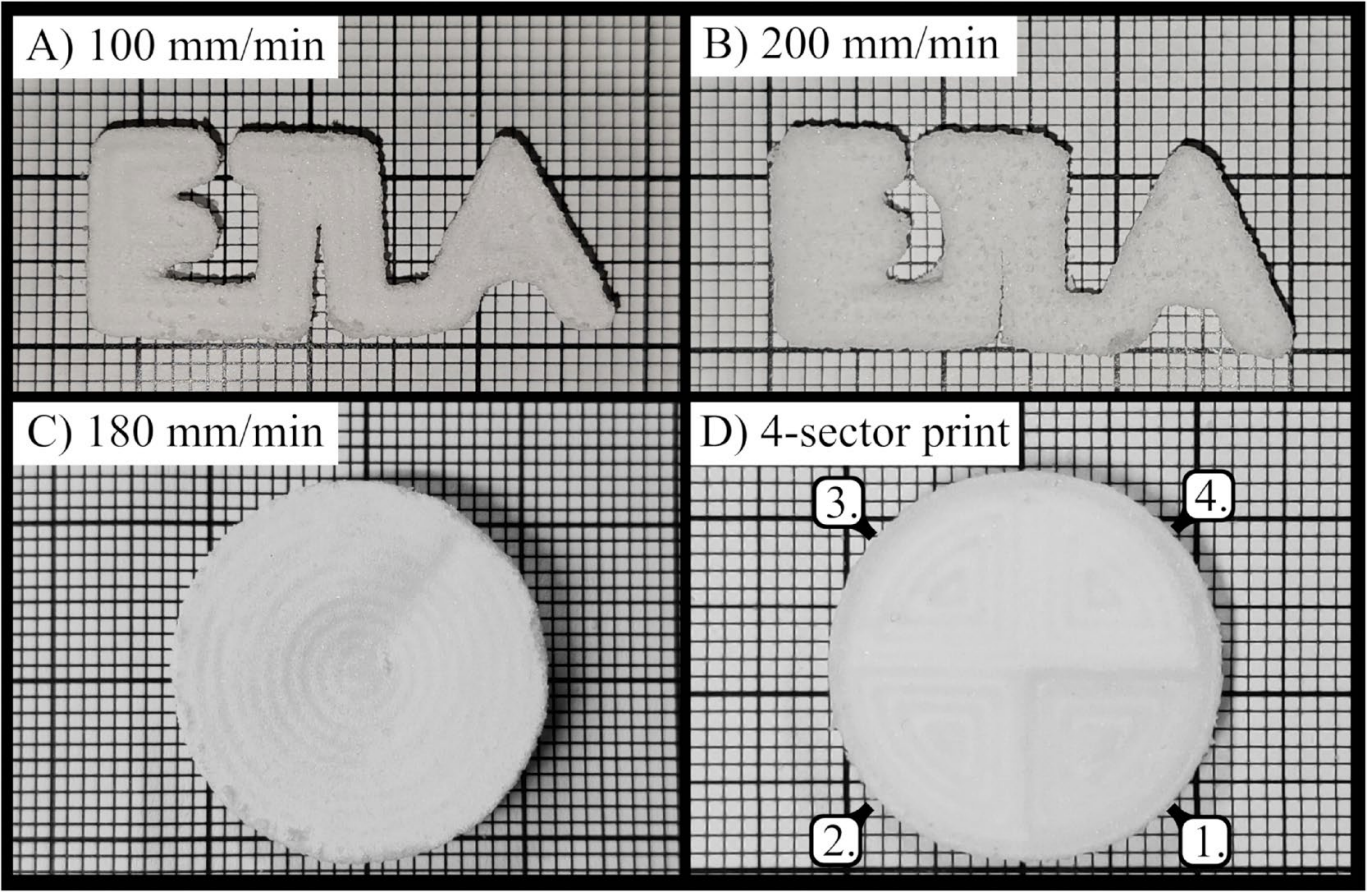
ETLA logos printed at a feed rate of $100\hbox { mm min}^{-1}$ (**A**) and $200\hbox { mm min}^{-1}$ (**B**) are shown in the top row. A disk with a single speed of $180\hbox { mm min}^{-1}$ (**C**) and a four sector disk (**D**) are shown in the bottom row. Speeds on the four sector disk are labeled from 1 to 4 with speeds of $25\hbox { mm min}^{-1}$, $75\hbox { mm min}^{-1}$, $125\hbox { mm min}^{-1}$ and $170\hbox { mm min}^{-1}$ respectively. The bold grid is $10\,\hbox {mm}$.

We printed structurally stable samples with repeatable print quality, achieving a maximum thickness of $5\,\hbox {mm}$. The printed ETLA logo at different feed rates as well as the samples for the cell tests, all produced at a constant transducer power, are shown in Fig. 1. Each sample had a thickness of $3\,\hbox {mm}$ and the printing took 5 to 10 min. All printed objects remained mechanically stable and intact after drying. The samples printed at different feed rates exhibited pronounced differences in mechanical properties: e.g. the ETLA logo printed at $100\hbox { mm min}^{-1}$ had a solid core and was rigid, whereas the $200\hbox { mm min}^{-1}$ print was brittle and highly porous. The individual printing lines are visible on the prints in Fig. 1 and their contrast is highlighted in supplementary figure S1.

### Simulation of the heat release in the focus

The peak acoustic power calculated from the measured hydrophone scan was 53.6 W, with an average acoustic power of 1.03 W. The measured attenuation coefficient and sound speed of the PCL-water suspension at a frequency of 4.2 MHz were $2.0\hbox { dB cm}^{-1}$ and $1760\hbox { ms}^{-1}$, respectively. The peak acoustic power and acoustic properties of the PCL-water suspension were used to model an equivalent piston focusing source in the *HIFU beam* software for simulating the nonlinear propagation of a radially symmetric ultrasound beam in a layered medium composed of absorbing materials.

**Fig. 2:**
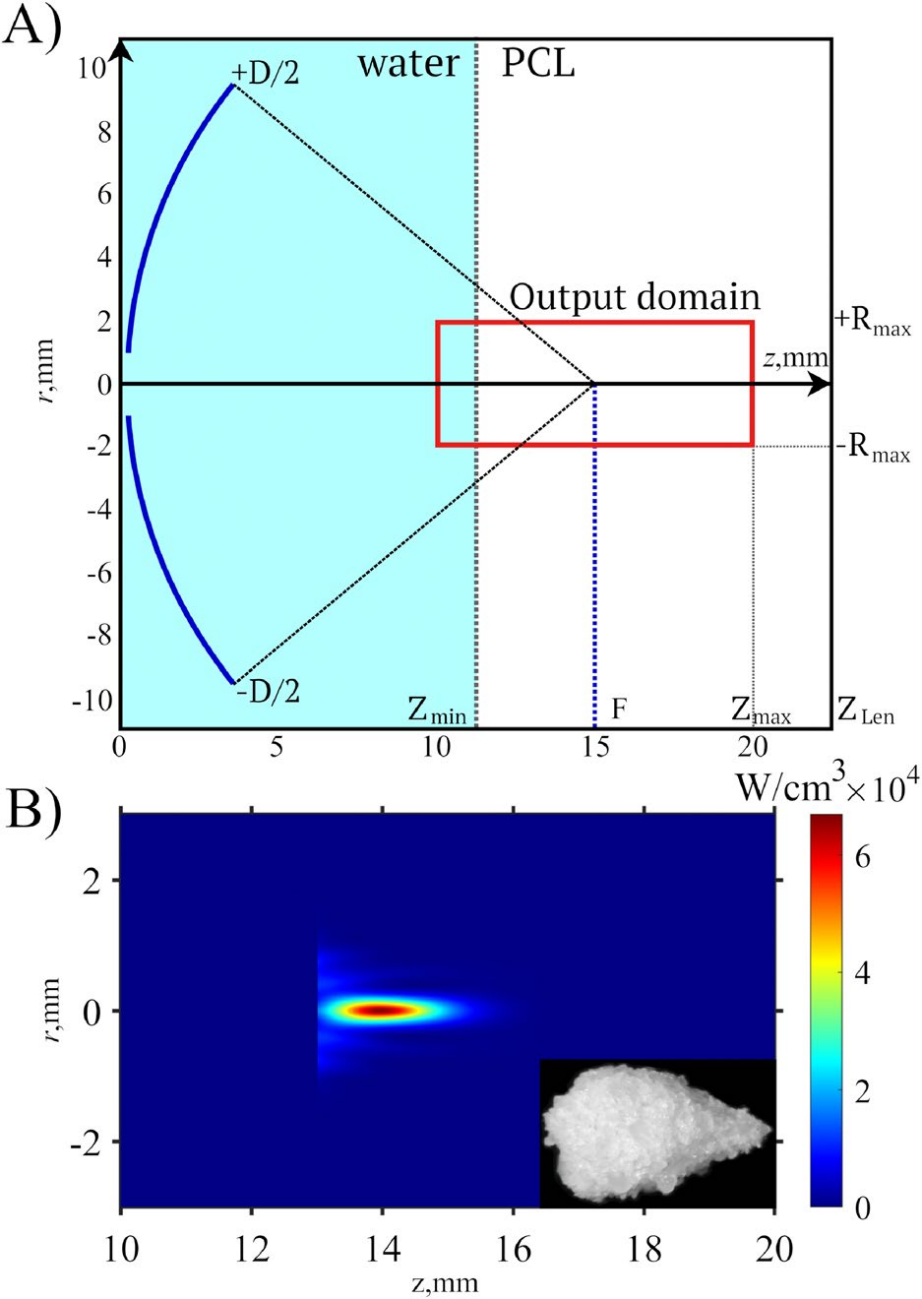
Geometry of the acoustic field simulation (**A**) and the simulated heat deposition in the focal area of the output domain (**B**). A scale matched five-second-sonicated point of PCL is shown in comparison to the focal area.

The geometry of the problem is shown in Fig. 2A. The medium consisted of two layers: water and the PCL-water suspension. The geometric focus of the transducer was located $2\,\hbox {mm}$ from the interface in the suspension layer.

The simulation showed that the acoustic intensity at the focus reached $2.3\;\hbox {kW/cm}^{2}$ with a corresponding negative peak pressure of 17.4 MPa (mechanical index of $8.5\,\hbox {MPa}\, \hbox{MHz}^{-0.5}$). The heat release distribution (Fig. 2B) has a maximum value of $63\;\hbox {kWcm}^{-3}$. At this level of heat power density, PCL begins to melt after 90 ms of sonication. A sample produced by sonicating the material at one point for 5 s is shown in the corner of Fig. 2B.

### Microstructure of the printed samples

**Fig. 3:**
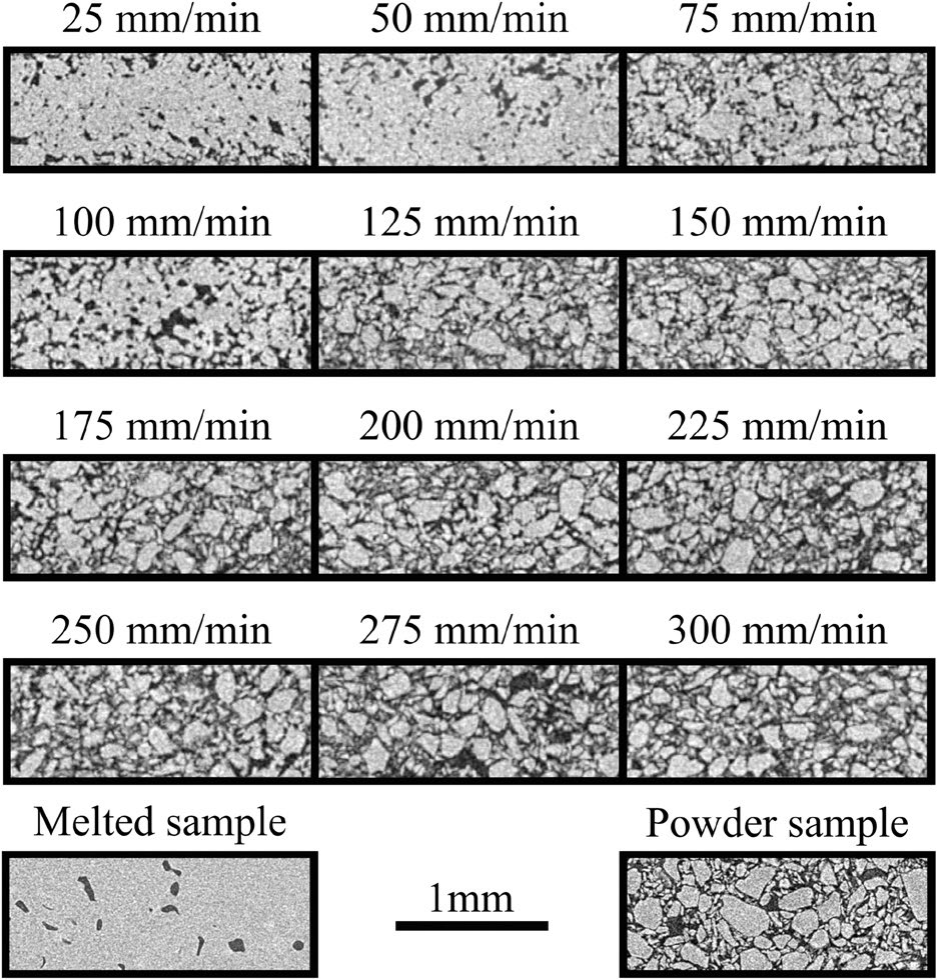
$\upmu$CT-imaged microstructure in printed PCL as a function of feed rate: $25\hbox { mm min}^{-1}$ to $300\hbox { mm min}^{-1}$. The structure of a thermally melted sample obtained by immersion in boiling water and an unsonicated powder sample are presented for reference. The voxel resolution of the $\upmu$CT scans is $15\,\upmu \hbox {m}$ for the different feed rates and $7.5\,\upmu \hbox {m}$ for the reference (melted and powder) samples.

Figure 3 shows $\upmu$CT images of the microstructure in the center of the samples printed at different feed rates. At feed rates up to $100\hbox { mm min}^{-1}$, the powder particles fused, forming large, interconnected structures. In contrast, at feed rates of $125\hbox { mm min}^{-1}$ and above, the material’s microstructure resembles that of the untreated reference sample. The melted sample was generated by immersing the encapsulated printing material into boiling water. This melts the entire sample at once which allows the separation of the polymer from the water.

**Fig. 4:**
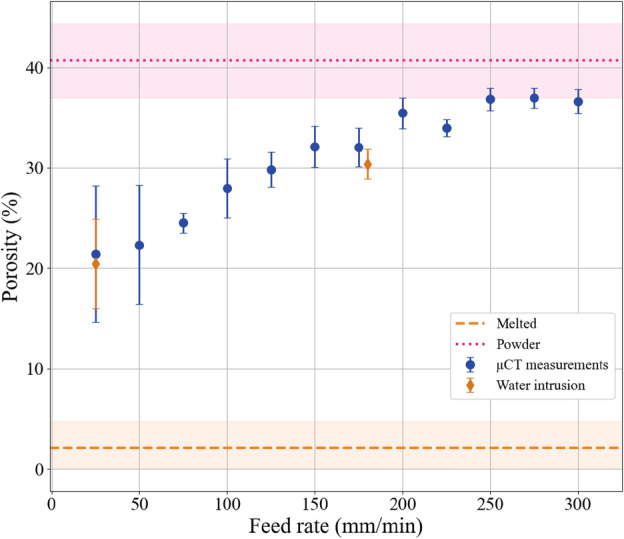
Porosity of PCL as a function of feed rate. The porosity of thermally melted PCL and dry powder are shown for reference. For each porosity value, the mean and one standard deviation are shown. Two additional data points from the water intrusion method are shown.

Figure 4 shows the PCL porosity in the samples calculated from the $\upmu$CT 3D scans. The decreasing porosity of PCL at low feed rates indicates that the powder is completely melted, leading to grain fusion and displacement of water from the samples. At high feed rates, the porosity approaches that of the untreated sample, indicating that the powder grains are connected only by tiny bridges formed by surface melting (not resolved in the $\upmu$CT images). The pores form a net-like structure with a characteristic transverse size of $10\,\upmu \hbox {m}$.

### Interconnection of pores

The interconnection of pores should enable nutritient exchange and potentially cell penetration into the printed scaffold, which is important for tissue engineering applications. Moreover, the pore size determines if cells can populate the scaffold.

The PCL volume porosity measurements for the feed rate of $25\hbox { mm min}^{-1}$ and $180\hbox { mm min}^{-1}$ obtained by the water intrusion method are consistent with those obtained by the $\upmu$CT method, Fig. 4. The agreement between the results obtained with both methods indicates that most pores in the samples are interconnected.

**Fig. 5:**
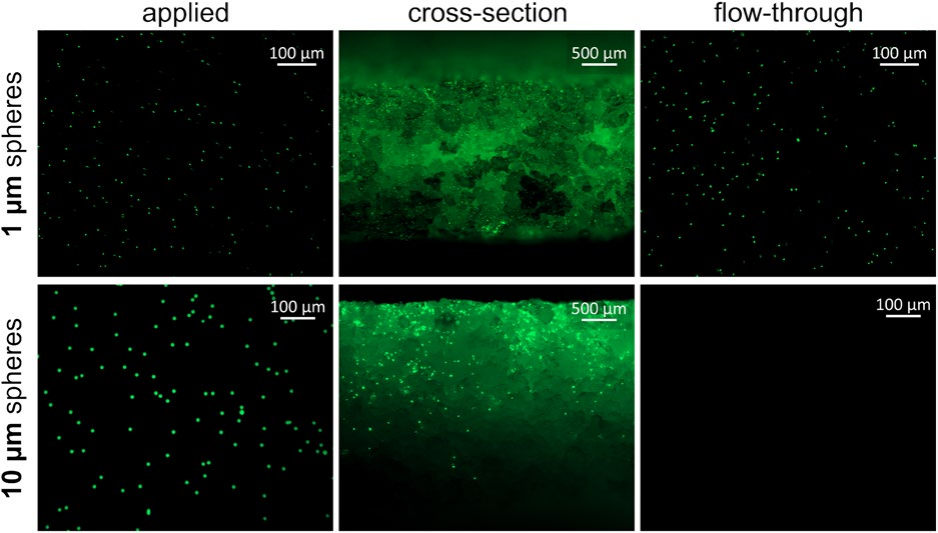
Microscope images of the applied suspension containing fluorescent microspheres, the scaffold cross-sections after applying the suspension, and the suspension that flowed through the sample. The samples printed at a feed rate of $300\hbox { mm min}^{-1}$ had an average thickness of $3\,\hbox {mm}$. Suspensions containing microspheres with diameters of $1\,\upmu \hbox {m}$ and $10\,\upmu \hbox {m}$ were applied on the top of the samples. The suspension permeated through the sample, carrying the microspheres into their internal structure.

Figure 5 shows sample cross-sections after passing water-containing microspheres through them. The $1\,\upmu \hbox {m}$ microspheres are observed throughout the entire depth of the sample, whereas $10\,\upmu \hbox {m}$ microspheres exhibit limited penetration, mainly accumulating on the surface and in the upper layers of the sample. Water containing both $1\,\upmu \hbox {m}$ and $10\,\upmu \hbox {m}$ spheres was visualized by microscope after passing through the sample (Fig. 5). The results confirm that the $1\,\upmu \hbox {m}$ spheres readily penetrate through the sample, whereas the $10\,\upmu \hbox {m}$ spheres are mainly trapped inside or on the surface of the sample.

### Cytocompatibility of the PCL scaffolds printed using SUM

**Fig. 6:**
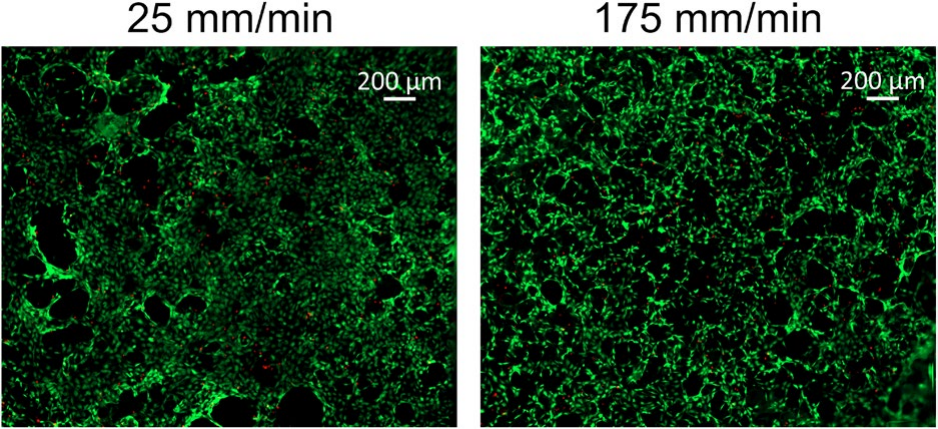
Cell viability and distribution on the scaffold with four-sector design. The sectors were printed with 25, 75, 125, and $175\hbox { mm min}^{-1}$ feed rates. NIH-3T3 fibroblasts were cultured on this scaffold for 3 days, followed by the live (green)/dead (red) staining.

PCL is commonly used in medical applications, hence the cytocompatibility of the printed objects was investigated. To understand whether the microstructure and porosity of the objects affect cell adherence and viability, the four-sector scaffold design (with 25, 75, 125, and $175\hbox { mm min}^{-1}$ feed rates) was used (Fig. 1D). Live/dead imaging by FDA/PI of fibroblasts cultured on these scaffolds showed > 90 % cell viability in all sectors. Representative images of 25 and $175\hbox { mm min}^{-1}$ sectors are shown in Fig. 6. More examples are presented in supplementary Fig.S2. Interestingly, the cells were equally distributed among the sectors, suggesting no preference for growing in a specific area.

### SEM images of cell-laden scaffolds

**Fig. 7:**
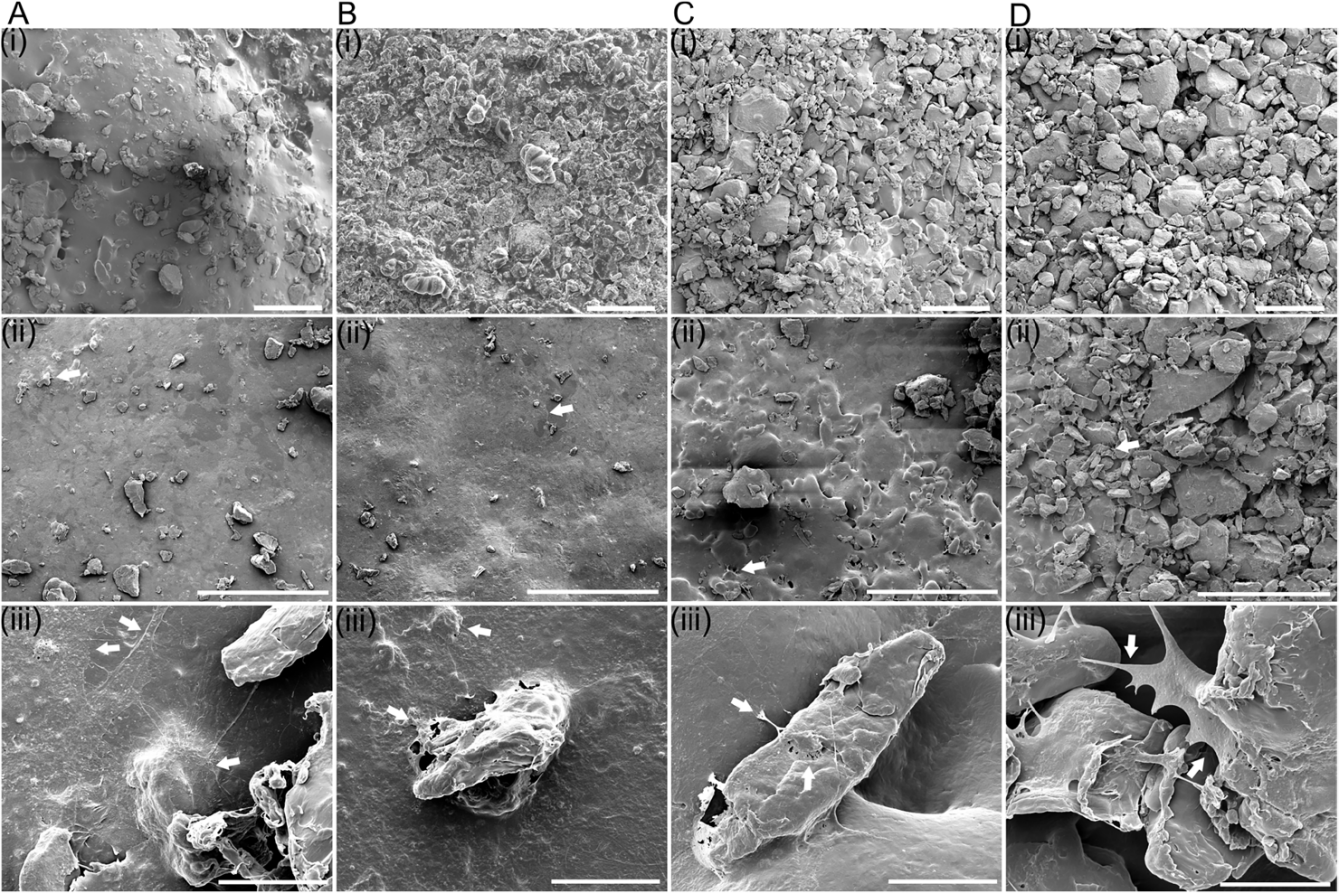
SEM images of the PCL scaffolds. Images showing surface topography of 3D scaffolds printed at different feed rates. (**A**) $25\hbox { mm min}^{-1}$, (**B**) $75\hbox { mm min}^{-1}$, (**C**) $125\hbox { mm min}^{-1}$, and (**D**) $175\hbox { mm min}^{-1}$. (i) Images showing control scaffolds not seeded with cells, (ii) and (iii) showing images of cells (white arrows) growing on the respective scaffold surface, either as monolayers or with 3D morphology. Scale bar- (i),(ii) $500\,\upmu \hbox {m}$, (iii) $20\,\upmu \hbox {m}$.

SEM analysis of the 3D scaffolds provides high-level information about the surface topography of the scaffolds printed at different feed rates. To analyse the surface properties, SEM analysis was done on the printed 3D scaffolds without cell seeding. The fusion of the PCL particles decreased with higher feed rates, leading to increased surface roughness (Fig. 7(i)). This translates into higher porosity of the scaffolds printed at high feed rates. In contrast, scaffolds printed at low feed rates (Fig. 7) A(i) and B(i) exhibited a higher degree of particle fusion and smoother surfaces. The SEM analysis of cell seeded scaffolds (Fig. 7(ii) and (iii)) demonstrated similar results. It was observed that at $25\hbox { mm min}^{-1}$ and $75\hbox { mm min}^{-1}$ feed rate the scaffolds have smoother surface, where the cells grow in a monolayer fashion with flat extended morphology. In contrast, the cells growing on the high feed rate scaffolds i.e. $125\hbox { mm min}^{-1}$ (Fig. 7C(ii, iii)) and $175\hbox { mm min}^{-1}$ (Fig. 7D(ii, iii)), demonstrate more 3D-morphology with anchoring filopodia. Formation of such structures by cells indicates cell proliferation on the scaffolds. Although PCL itself is hydrophobic and hence do not feature cell anchoring motifs, serum treatment of the scaffolds might lead to surface protein adsorption enabling cellular adhesion and proliferation on the printed scaffolds. Similar results were observed with NaOH treated scaffolds (Fig. S4). Here prominent cell adhesion to the scaffold surface, either as monolayers or with 3D extendend morphology were observed. In both cases, the results show cell adhesion and proliferation with no significant difference in the cytocompatibility of the scaffolds.

## Discussion

Printing PCL samples using SUM offers unique advantages. Due to its biodegradability and slow degradation rate, PCL is valued in biomedical applications, including tissue engineering, regenerative medicine, and drug delivery^26^.

The adjustment of porosity provides an effective way to control the degradation rate of PCL, with higher porosity resulting in accelerated weight loss of the material^14^. Furthermore, controlling the porosity and connectivity of particles allows tuning the mechanical properties of the scaffold, while optimizing the surface roughness improves its biological performance^27^.

Further customization of the structural, mechanical, surface, and biocompatible properties of the samples can be achieved by incorporating additional components into the base material and by selecting different liquids for the suspension preparation. The bulk mechanical properties of the printed samples can be tailored by adding other materials that are blendable with PCL such as polymers, starch, lignin, etc.^28,29^. For example, incorporating silica aerogel into PCL results in modified properties suitable for use in scaffolds for artificial bone^30,31^. Adjusting the particle size and shape of the powder grains can lead to changes in structural characteristics, such as increased porosity or larger pore size. Additionally, replacing water with other aqueous substances enables further tuning of material properties. For example, embedding nutrients directly into the material can promote cell growth.

PCL-based material used for SUM printing offers advantages over silicone rubber^5^. Silicone rubber requires mechanical separation of the printed object from the soft, sticky uncured material, such rubber has a limited pot life that constrains print time, and generates waste since it can not be reused. In contrast, PCL avoids these limitations since it is stable and can be reused.

Resolution is a key factor determining print quality, which is mainly affected by the power and frequency of the acoustic waves, the printer’s feed rate, and the suspension’s acoustic properties. Both feed rate and acoustic power affect the amount of acoustic energy per mass of material focused into the printing area. This energy is eventually converted into heat, which diffuses and reduces print resolution as more energy is delivered. Increasing the frequency can improve resolution, since high frequencies create a small focal size. However, high frequencies increase the acoustic attenuation which limits penetration depth and restricts the maximum thickness of printed objects. When selecting the operating frequency the wavelength in the material should be significantly larger than the grain size in the suspension to prevent scattering based attenuation^32^. In the current study the maximum thickness of the printed samples was $5\,\hbox {mm}$. Thus using suspensions with smaller grain size can improve the resolution. For maximum resolution, the acoustic absorption in the material should be as small as possible, allowing the material to be heated primarily by nonlinear effects. Focusing high intensity ultrasound into very small volumes allows for localized and effective heating^33^. Therefore, an optimal approach to enhancing print resolution involves minimizing attenuation by employing low frequencies, reducing the grain size of the powder, and by applying short, high amplitude bursts to achieve highly localized heat deposition.

The patterns of the prints (Fig. 1) with visible printing paths resemble the patterns of objects produced using FDM printing. Similarly to FDM printing, the mechanical strength of ultrasonically printed samples is influenced by the slicing parameters, particularly the spacing between the printing paths. The line separation distance determines the degree of fusion between adjacent paths in both horizontal and vertical directions. Similarly to FDM printing, where the extruded filament thickness is defined by the translation speed and extrusion rate; in ultrasonic printing, the molten path thickness is determined by the feed rate and acoustic power. Figure 1C illustrates the variation in the thickness of the printing path for different feed rates at constant acoustic power. At the lowest feed rate, the molten path (darker regions) is thick and nearly fused with the adjacent paths, though a small amount of partially fused powder (lighter regions) remains between the paths. At high feed rates, the molten path becomes thinner and the presence of unmelted powder between the printing paths becomes more pronounced. Unmelted parts reduce the overall structural integrity of the sample. Thus, unlike FDM printing, an ultrasonically printed sample consists not only of molten paths but also includes partially fused material connected to these paths.

The experiments with the fluorescent microspheres confirmed that the pores are interconnected and that particles with at least a size of $1\,\upmu \hbox {m}$ can permeate in the printed object. The $10\,\upmu \hbox {m}$ microspheres were unable to penetrate through the print done at $300\hbox { mm min}^{-1}$. Thus, the typical pore size in unfused samples (printed at high feed rates) is between 1 and 10 μm. The pore size would be expected to be smaller with slow feed rates when considering that the porosity of PCL increases with increasing feed rates as seen in Fig. 4.

NIH-3T3 fibroblasts seeded on the printed PCL scaffolds were attached and proliferated, which indicates that the scaffolds are cytocompatible (Figs. 6, 7, S2 and S5). Of note, NIH-3T3 cells can be maintained under standard cell culture conditions using different serums, specifically, calf serum or fetal bovine serum (FBS)^34,35^. However, in our experiments the serum choice turned out to be crucial for the survival of these cells in case they were seeded on the PCL scaffolds (Fig.S3). NIH-3T3 cell viability remained high when they were cultured with the calf serum, but not with the FBS. Interestingly, coating the PCL surface could improve both cell attachment and proliferation on it. For example, poly-D-lysine and collagen type-I coating were used previously^25^. Taking into account that calf serum and FBS have different compositions, it remains to be investigated which components of calf serum improved the NIH-3T3 cell attachment.

Another PCL surface modification method is sodium hydroxide (NaOH) etching, which increases the hydrophilicity of the PCL scaffold, and thus, cell attachment to it^36^. However, treatment of the ultrasound-printed scaffolds with 5M NaOH for 30 min did not influence the ability of NIH-3T3 fibroblasts to grow on those scaffolds (Fig. S4). The high resolution SEM imaging of the 3D printed scaffolds demonstrated a correlation between feed rate and the porosity of the printed scaffolds. Slow feed rates demonstrated high PCL particle fusion and material consolidation, leading to a smooth printed surface. This leads to low structural porosity of the printed scaffolds. Such low porosity structures may provide high mechanical strength to the structure but the lack of macro-porosity hinders cell penetration into the structure where they were mostly growing as monolayers on the surface. Alternatively, high printing feed rates lead to low particle fusion with much higher surface roughness and structural porosity in the printed scaffold. With the SEM analysis cells were observed to grow in a more 3D morphology on these scaffolds with formations of extended cell adhesion appendages. Such porous structures provide interconnected macroporous architectures for cell penetration and are desired in certain tissue engineering applications. With a tuned porosity the high surface area for cell growth and inter-connectivity for mass transfer of nutrients, fluids and gaseous exchange could be provided which are required to sustain cell proliferation. The structural porosity can be tuned with SUM printing and it should be explored in further studies. The confirmed cell attachment and proliferation demonstrated the absence of negative effects to cytocompatibility on the PCL arising due to SUM printing.

In the current study, cell migration into the samples was not observed (data not shown). This could be due to either insufficient cell culture time or too small pore size. To address this issue, the pore size could be increased by using a coarser powder combined with an optimized print feed rate. This would tailor the microstructure of the samples to support cell growth within the structure better. The minimum value for efficient cellular infiltration of fibroblast has been identified to be $10\,\upmu \hbox {m}$ while the optimal size would be around $25\,\upmu \hbox {m}$ to $60\,\upmu \hbox {m}$^37^. Since most of the $10\,\upmu \hbox {m}$ diameter microbeads were trapped on the surface of the printed scaffold as shown in Fig. 5, the pore size is is lower than the optimal but close to the minimum threshold for cell infiltration.

In addition to pore dimensions, architecture and interconnectivity, the cell migration into a scaffold also depends on factors such as mass flow, nutrient transport and vascularization within the scaffold. It has been observed that skin fibroblast effectively migrated in scaffolds having pores in rage of 20 $\upmu \hbox {m}$ to 125 $\upmu \hbox {m}$^38^. However, further studies have shown the pores sizes ranging between 5 and 18 μm is considered ideal for bioengineered skin graft scaffolds for effective attachment of keratinocytes and fibroblasts. It has been observed that scaffolds porosity, pore size distribution and interconnectivity strongly influence cell migration within such bioengineered scaffolds^39,40^.

During the printing, in addition to the thermal effects on PCL, cavitation occurs. Cavitation can form chemical species^41^, some of which may be toxic to cells. Simulations of the acoustic field propagation revealed a negative pressure value of 17.4 MPa at the focus, corresponding to a mechanical index (MI) of $8.5\;\hbox{MPa}\;\hbox{MHz}^{-0.5}$ at an operating frequency of 4.17 MHz. High MI values indicate a high probability of cavitation, which typically occurs at MI values above 0.4, suggesting that cavitation occurred during printing. Despite this, the results of this work demonstrated high cytocompatibility of the printed samples, indicating that the cavitation process did not negatively affect cytocompatibility. These findings align with a study^42^, which reported no toxic effects on cells from chemical species formed during cavitation in PCL diol.

Reduction of MTT (3-(4,5-dimethylthiazol-2-yl)-2,5-diphenyl-2H-tetrazolium bromide) reagent to formazan product is a widely used colorimetric method to assess the metabolic activity of cells^43^. Such a reaction is supposed to occur only in living cells, hence, the cell viability can be determined. Therefore, it was unexpected to observe a substantial background and formation of formazan crystals when the PCL powder alone or cell-free scaffolds were incubated with the MTT solution. FDA/PI staining was used as a mitigation strategy.

## Conclusion

We presented the selective ultrasonic melting of PCL powder by using a focusing piezo transducer and a high-power piezo driver. The transducer was attached to a CNC machine to scan the focal point through a suspension of PCL and water. Prints were performed at an average acoustic power of 1.03 W. Scanning with feed rates from 25 to $300\hbox { mm min}^{-1}$ resulted in solid porous objects.

The proposed method of acoustic 3D printing outperforms the previous method with silicone rubber with regards to reusability, biodegradability, and potential for future applications. The presented method provides an alternative to methods such as FDM and binder jetting. With PCL established in tissue engineering and regenerative medicine, the proposed approach provides a new method for fabricating scaffolds. The outlined method of selective acoustic melting allows control of porosity by varying the feed rate which was confirmed with microstructure analysis using $\upmu$CT and SEM. Using fluorescent microspheres it was confirmed that the structures possess an interconnected pore architecture, which can be tuned by altering the print parameters. The live-dead analysis and SEM imaging confirmed cyto-compatibility of the printed structures, which is beneficial for biomedical applications. This also indicates that the fabrication method does not generate toxic by-products.

## Methods

### Ultrasonic 3D printer implementation

**Fig. 8:**
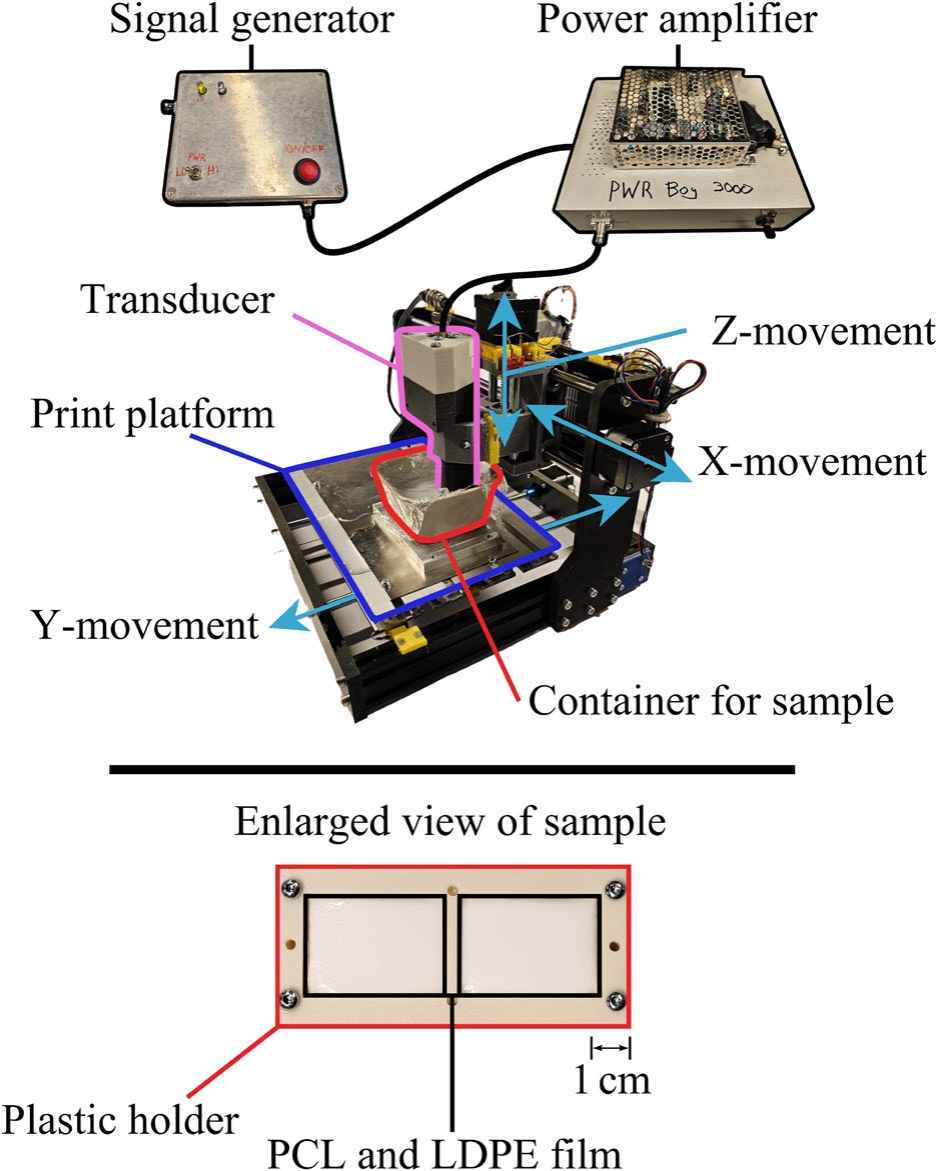
The upper part illustrates the experimental setup, comprising the signal generator, power amplifier, and ultrasonic transducer mounted on the translation stage, allowing the transducer to move relative to the sample. The lower part shows the sample holder, which keeps the PCL-water suspension sample enclosed in a resealable LDPE bag.

The custom made SUM device comprises an electrical signal generator, an acoustic transducer, and a mechanical translation system. A schematic overview is shown in Fig. 8.

The electric signal was generated by a high-power piezo-driver which uses a single-ended MOSFET topology^44^. This MOSFET produces a high-current signal which is transformed into a high-voltage signal by a transformer, that adjusts the output impedance to $50 \;\Omega$. A low-pass filter suppresses higher harmonic components at the output. The MOSFET is driven by a low-voltage logic level square-wave burst. For the experiments, the signal parameters were set at 80 cycles, with a frequency of 4.17 MHz, 50 % duty cycle, and a pulse-repetition-frequency of 1 kHz. This configuration produced sinusoidal bursts with 79 V amplitude. The average electric power consumption of the piezo driver during signal generation was 1.2 W, with a peak power of 62.4 W.

An ultrasonic focusing transducer was built from a piezo bowl (CTS Ferroperm, Denmark) with a radius of curvature of $15\,\hbox {mm}$, aperture of $\varnothing 19\,\hbox {mm}$, central hole diameter of $\varnothing 4\,\hbox {mm}$, and resonance frequency of 4.17 MHz. The transducer enclosure was 3D-printed, and the piezo bowl was glued to it with epoxy resin. A built-in matching circuit in the transducer adjusted the transducer’s resistance to $50 \;\Omega$ at the resonance frequency.

The transducer was mounted on a Genmitsu 3018 3-axis CNC machine (Sainsmart, USA) for positioning. The printing path was programmed using G-Code that was loaded onto the CNC machine. The transducer’s translation speed along the path (feed rate) was varied in from 25 to $300\hbox { mm min}^{-1}$.

### Printing material

The printed material was prepared from medical-grade PCL powder (Magerial Science, USA), with a number average molecular weight of $50\hbox { kg mol}^{-1}$ and a maximum particle size of $149\,\upmu \hbox {m}$ (mesh size 100). PCL is insoluble in water and has a melting point of around 60 °C^45^. The powder was mixed with deionized, degassed water at a weight ratio of 3:2 to form a suspension, which was further degassed to remove trapped air. Excess water was then removed forming a 2:1 PCL-water suspension. The suspension was placed in an acoustically transparent resealable bag made of low-density polyethylene (LDPE) film with a thickness of $45\,\upmu \hbox {m}$. The bag was fixed with a plastic holder and immersed into a container of degassed water which served as a sonic coupling medium between the sample and the acoustic transducer.

### Acoustic field characterization

The acoustic field generated by the transducer for printing was characterized using a calibrated fiber-optical hydrophone (Onda, USA) in a plane transverse to the transducer axis positioned $5\,\hbox {mm}$ from its surface. The aperture of the hydrophone was $100\,\upmu \hbox {m}$ and the bandwidth ranged from 3 kHz to 150 MHz. The scanning was performed in the near-field as cavitation is present in the focal area. A $1\times 1\,\hbox {cm}^{2}$ area was scanned with a $200\,\upmu \hbox {m}$ step size, recording the full waveform at each point. Based on the scan data, the characteristics of the acoustic beam were calculated, including acoustic power and the vibrational velocity of the transducer surface^46^.

The measured characteristics of the acoustic beam were used as input parameters for the *HIFU beam* software^47^. The sound speed and attenuation coefficient of the PCL suspension, necessary for the simulation, were measured using the insertion loss method^48^. The *HIFU beam* software was used to calculate the acoustic pressure, acoustic power, and thermal power in the focal area. These values allowed to estimate the sonication time required to melt the material as well as to determine the printing resolution.

### Micro computed tomography analysis

Micro computed tomography ($\upmu$CT) was used to investigate the micro-structure of the prints. Scans were conducted with the Skyscan 1272 (Bruker, USA) high-resolution 3D X-ray scanner with a voxel resolution of $7.5\,\upmu \hbox {m}$ and $15\,\upmu \hbox {m}$. This method allows for non-destructive visualization of the printed 3D microstructure, as well as measurements of variations in material density and porosity. *Fiji Imagej* software^49^ was used to process CT data to calculate the porosity of the samples.

### Assessment of open porosity

The Water intrusion method^50^ was used to assess the open porosity of the material. Four disk shaped samples with a height of $4\,\hbox {mm}$ and a diameter of $22.5\,\hbox {mm}$ were printed at a feed rate of $180\hbox { mm min}^{-1}$. The samples were dried at room temperature for several days with their weights monitored using an analytical 410AM-FR balance (Precisa Gravimetrics AG, Switzerland) until a constant weight was achieved, at which point the samples were considered dry. Subsequently, they were immersed in deionized water at room temperature and degassed for 30 min to remove entrapped air. The samples were then taken out of the water and excess water was removed. The bulk porosity of the PCL scaffolds was calculated from the measured weights and known densities.

The sizes of the pore connections were evaluated using fluorescent microspheres (Polysciences, USA) with diameters of $1\,\upmu \hbox {m}$ and $10\,\upmu \hbox {m}$. The microsphere suspension was diluted with water at a volume ratio of 1:200 and pipetted on top of each sample. The passed-through liquid was optically analyzed using the Axio Zoom.V16 fluorescent microscope (Zeiss, Germany) equipped with an HXP 120 V light source (LEJ, USA). The samples were cut in half to obtain cross-sectional images to evaluate the penetration of the microspheres. Fluorescent images were analyzed using ZEN 3.8 software (Zeiss, Germany).

### Live/dead imaging to assess cell viability

A four-sector cell culture scaffold was designed, with each sector printed at different feed rates: 25, 75, 125, and $175\hbox { mm min}^{-1}$. Printing all feed rates in one sample minimized the potential impact of batch-to-batch variation in the printed material and reduced risks of microbiological contamination.

Murine NIH-3T3 fibroblasts (CRL-1658, ATCC) were cultured under standard conditions (37 °C, 5 % CO_2_) in Dulbecco’s Modified Eagle Medium (DMEM, high glucose, $\hbox {Glutamax}^{\textrm{TM}}$ Supplement, pyruvate; $\hbox {Gibco}^{\textrm{TM}}$, Thermo Fisher Scientific Inc., Waltham, USA) supplemented with 10 % calf serum (iron supplemented, US origin, $\hbox {Hy-clone}^{\textrm{TM}}$), 1 % $100\hbox { IU mL}^{-1}$ penicillin and $100\, \upmu\hbox {g mL}^{-1}$ streptomycin ($\hbox {Gibco}^{\textrm{TM}}$). The cells were passaged 2–3 times a week at the subculture ratio of 1:10 - 1:15 using $\hbox {Tryple}^{\textrm{TM}}$ Express Enzyme ($\hbox {Gibco}^{\textrm{TM}}$).

The printed scaffolds were sterilized with 70 % ethanol for 1 h, washed 3 times with Dulbecco’s phosphate buffered saline (DPBS, no calcium, no magnesium, $\hbox {Gibco}^{\textrm{TM}}$), and soaked in the calf serum for 1 h. Then the scaffolds were placed in the complete DMEM medium supplemented with $0.25\,\upmu \hbox {g mL}^{-1}$ amphotericin b and $10\,\upmu \hbox {g mL}^{-1}$ gentamicin ($\hbox {Gibco}^{\textrm{TM}}$) to inhibit potential fungal or bacterial contamination and left in the incubator overnight.

The next day, $1.5 \times 10^{5}$ NIH-3T3 cells were seeded on the scaffolds and cultured in a 12-well plate. The cells cultured in the well without a scaffold were used as a positive control to verify the viability of the cells. After 3 days, the cell-containing scaffolds were stained with $8\,\upmu \hbox {g mL}^{-1}$ fluorescein diacetate (FDA, BLD Pharmatech Ltd., Shanghai, China) and $20\,\upmu \hbox {g mL}^{-1}$ propidium iodide (PI, BLD Pharmatech Ltd.) dissolved in DMEM medium supplemented with 2 % calf serum. The staining was done at room temperature in the dark for 5 min followed by two washings with DPBS. The live and dead cells were imaged with a fluorescence microscope (Zeiss Axio Zoom.V16, Germany). The z-stack images were analyzed with the ZEISS ZEN 3.8 software.

Cell viability was calculated based on the live/dead images according to a published protocol^51^ for automatic quantification of live and dead cells using Fiji-Imagej.

### SEM images of cell-containing scaffolds

After verifying the presence of the cells on the scaffolds by live/dead imaging, the same cell-containing scaffolds were fixed with the paraformaldehyde solution (4 % w/v) in $0.2\hbox { mol L}^{-1}$ sodium cacodylate buffer pH 7.2 for 4 h at 4 °C. The samples were rinsed with PBS followed by deionised water. The samples were then dehydrated via step-wise sequential ethanol gradient method, starting from 30 % v/v to 100 % v/v with a hold time of 30 min at each step. The samples finally dried by placing in a desiccator for 24 h to remove traces of moisture. Before imaging, the samples were sputter coated with 5 nm of platinum. The SEM imaging was performed using FEI Quanta-250 (FEI, Hillsboro, USA). The control 3D-scaffolds were processed in similar manner for SEM imaging to analyse surface topography, however they were not seeded with cells.

## Data Availability

Data is provided within the manuscript or supplementary information files. Datasets generated during the current study are available from the corresponding author on reasonable request.
